# Changes in the proteome of the problem weed blackgrass correlating with multiple‐herbicide resistance

**DOI:** 10.1111/tpj.13892

**Published:** 2018-04-25

**Authors:** Catherine Tétard‐Jones, Federico Sabbadin, Stephen Moss, Richard Hull, Paul Neve, Robert Edwards

**Affiliations:** ^1^ Agriculture, School of Natural and Environmental Sciences, Newcastle University Newcastle upon‐Tyne NE1 7RU UK; ^2^ Department of Biology University of York York YO10 5DD UK; ^3^ Stephen Moss Consulting 7 Alzey Gardens Harpenden Hertfordshire AL5 5SZ UK; ^4^ Rothamsted Research Harpenden Hertfordshire AL5 2JQ UK

**Keywords:** abiotic and biotic stress, *Alopecurus myosuroides*, multiple drug resistance, safeners, transcriptomics

## Abstract

Herbicide resistance in grass weeds is now one of the greatest threats to sustainable cereal production in Northern Europe. Multiple‐herbicide resistance (MHR), a poorly understood multigenic and quantitative trait, is particularly problematic as it provides tolerance to most classes of chemistries currently used for post‐emergence weed control. Using a combination of transcriptomics and proteomics, the evolution of MHR in populations of the weed blackgrass (*Alopecurus myosuroides*) has been investigated. While over 4500 genes showed perturbation in their expression in MHR versus herbicide sensitive (HS) plants, only a small group of proteins showed >2‐fold changes in abundance, with a mere eight proteins consistently associated with this class of resistance. Of the eight, orthologues of three of these proteins are also known to be associated with multiple drug resistance (MDR) in humans, suggesting a cross‐phyla conservation in evolved tolerance to chemical agents. Proteomics revealed that MHR could be classified into three sub‐types based on the association with resistance to herbicides with differing modes of action (MoA), being either global, specific to diverse chemistries acting on one MoA, or herbicide specific. Furthermore, the proteome of MHR plants were distinct from that of HS plants exposed to a range of biotic (insect feeding, plant–microbe interaction) and abiotic (N‐limitation, osmotic, heat, herbicide safening) challenges commonly encountered in the field. It was concluded that MHR in blackgrass is a uniquely evolving trait(s), associated with changes in the proteome that are distinct from responses to conventional plant stresses, but sharing common features with MDR in humans.

## Significance Statement

We have examined changes in protein expression that are associated with multiple herbicide resistance (MHR) in blackgrass, that now threatens the sustainability of winter wheat production in Europe. The MHR proteome was distinct from that determined following exposure to environmental challenges, but showed several similarities to multiple drug resistance in human tumors. The results provide further insight into the molecular basis and evolution of a type of resistance that increasingly threatens global food security.

## Introduction

Herbicide resistance in weeds is now a global problem threatening the sustainable intensification of agriculture, notably in arable crops (Gressel, 2009; Busi *et al*., 2013; Délye *et al*., 2015). In the UK, herbicide resistance in blackgrass (*Alopecurus myosuroides*) affects 20 000 farms and costs the country £0.5 billion in lost winter wheat production (Hull *et al*., 2014). Two types of herbicide resistance are recognized. Target‐site resistance (TSR) is conferred by mutations in genes encoding enzymes targeted by herbicides, such as acetyl CoA carboxylases (ACCase) and acetolactate synthase (ALS). Such mutations give rise to enzymes that, while still functional, show a reduced binding affinity for herbicides. TSR is a well characterized resistance mechanism, classically selected through the repeated use of herbicides sharing a mode of action (Neve *et al*., 2014). Herbicide resistance can also arise through a more complex and less well understood group of mechanisms collectively termed non‐target‐site resistance (NTSR).

NTSR evolves as a multigenic quantitative trait and results in tolerance to most classes of herbicides used for post‐emergence weed control presently used in wheat (Neve *et al*., 2014). First reported in a single field in Peldon, Essex in 1984, NTSR in blackgrass is now established in the majority of blackgrass populations in the UK, often co‐existing with TSR (Hull *et al*., 2014). The mechanisms underpinning NTSR are poorly understood but include the reduced uptake and enhanced metabolism of herbicides and cytoprotection against the downstream effects of chemical injury (Busi *et al*., 2013; Délye *et al*., 2015). Of these mechanisms, NTSR is most commonly associated with enhanced herbicide metabolism. This requires the enhanced expression of multiple classes of detoxifying enzymes, notably cytochrome P450s (CYPs), glutathione transferases (GSTs) and transmembrane transporter proteins, a detoxification system we have collectively termed the ‘xenome’, having previously demonstrated the upregulation of multiple components in NTSR blackgrass (Cummins *et al*., 1999, 2013).

While NTSR is driven by repeated herbicide selection, it is also clear that this complex trait can be influenced by environmental conditions (Yuan *et al*., 2007; Délye, 2013; Vila‐Aiub *et al*., 2013). A plausible explanation for its rapid evolution would be a close mechanistic association between NTSR and responses to biotic and abiotic stress. There has been particular interest in the linkages between NTSR and the responses of grasses to treatment with herbicide safeners (Duhoux *et al*., 2017). Safeners are active components of herbicide formulations used in selective weed control that enhance chemical tolerance in cereals by accelerating their metabolism in the crop through xenome gene induction (Kraehmer *et al*., 2014). As such safening and NTSR both result in enhanced herbicide tolerance through the upregulation of detoxification systems. NTSR is also known to be associated with changes in gene expression in blackgrass (*Alopecurus mysosuroides*) (Gardin *et al*., 2015), annual rye‐grass (*Lolium rigidum*) (Gaines *et al*., 2014), barnyardgrass *(Echinochloa crus‐galli)* (*Yang et al.,* 2013), horseweed (*Conyza canadensis*) (Peng *et al*., 2010), and waterhemp (*Amaranthus tuberculatus*) (Riggins *et al*., 2010). In the case of *L*. *rigidum*, association transcriptomics suggested that only a limited number of these genes were causatively linked to NTSR resistance (Gaines *et al*., 2014).

While transcriptomics is a powerful tool to study changes in global gene expression associated with stress tolerance, it cannot always predict functional changes in plant metabolism. To characterize the mechanisms of herbicide resistance in greater detail we have instead examined the proteomes of several populations of NTSR blackgrass that have either evolved in the field through current practices of weed management, or have been generated in the lab through repeated selection with a single herbicide. In each case, we have compared these proteomes with those determined in untreated herbicide sensitive (HS) blackgrass and looked for proteins that are consistently associated with NTSR. In addition, we have compared the NTSR proteome with that obtained in HS plants exposed to a range of biotic and abiotic treatments to test the hypothesis that herbicide resistance is functionally linked to tolerance to stresses encountered under adverse field conditions.

## Results

### Generation and characterization of herbicide‐resistant populations of blackgrass

Blackgrass seeds collected from Section 8 of the Broadbalk trial site at Rothamsted were used as the HS reference population. This section has never received any herbicides since the experiment was established in 1843 (Hall *et al*., 1995). The Peldon and Oxford (Oxford S in previous publications) populations, previously characterized for their resistance to both ALS and ACCase acting herbicides (Hall *et al*., 1995; Moss *et al*., 2003), were used as the field‐derived NTSR populations.

In addition, Rothamsted HS plants were repeatedly selected for resistance to the herbicides fenoxaprop‐ethyl (over six generations) and pendimethalin (over eight generations) respectively (Table S1). These herbicides were used because whereas resistance to fenoxaprop can arise through both TSR‐ and NTSR‐based mechanisms (Cocker *et al*., 1999), evolved tolerance to pendimethalin in blackgrass is most commonly associated with enhanced detoxification (James *et al*., 1995). The progeny of the survivors from the selection studies were assessed for resistance to herbicides acting on ACCase (fenoxaprop, clofinafop, cycloxydim), ALS (mesosulfuron + iodosulfuron, sulfometeturon, pyroxsulam), tubulin assembly (pendimethalin) and fatty acid elongation (flufanacet), as detailed in Table S2. In each case, the blackgrass samples were assessed for herbicide resistance (Marshall *et al*., 2013), with the results presented in Table S3. The population selected for resistance to pendimethalin, showed cross‐resistance to fenoxaprop, clodinafop and cycloxidim that all act on ACCase, but not toward herbicides that inhibited ALS, or cell division (Table S3). PCR of the respective ACCases in the selected population (Marshall *et al*., 2013), confirmed that resistance was not due to TSR‐related mechanisms. In contrast, at the application rates tested, the population isolated following repeated selection with fenoxaprop was only resistant toward the selecting herbicide, showing no cross‐resistance to other ACCase inhibitors, or the other classes of herbicides tested. PCR of the ACCases present in the resistant plants showed no evidence of TSR, suggesting that a fenoxaprop‐specific NTSR mechanism had been selected for; a phenomenon previously determined in field populations exposed to this herbicide (Cocker *et al*., 1999). From these experiments, it was concluded that the pendimethalin‐ and fenoxaprop‐selected populations were exhibiting different sub‐classes of NTSR and that both were distinct from the more comprehensive herbicide cross‐resistance determined in the Peldon and Oxford field‐sourced resistant populations (Hall *et al.,* 1995; Moss *et al*., 2003; Marshall *et al*., 2013).

### Whole transcriptome analysis of NTSR blackgrass

To examine the potential for multiple mechanisms to underpin NTSR, the well characterized blackgrass population Peldon, that is known to be resistant to herbicides acting on ALS, ACCase, tubulin assembly and photosystem II (James *et al*., 1995; Cocker *et al*., 1999; Marshall *et al*., 2013), was selected as possessing a comprehensive multiple resistance phenotype. The Rothamsted population was then used as a reference HS population. cDNA libraries were prepared in biological triplicates from Peldon and Rothamsted and subjected to global RNA‐Seq analysis using the IonTorrent sequencing platform. The reference transcriptome created was then used to compare quantitative and qualitative differences in the cDNAs isolated from the two sets of samples. The methodology involved assembling overlapping RNA sequences (contigs), to construct Unigenes corresponding to the transcriptome of HS and NTSR plants respectively. For Unigenes identified in both samples, relative transcript abundance was then calculated as the fold difference in the number of contigs in the NTSR, as compared to the HS population. In total, 383 149 contigs were assembled.

The first obvious difference in the two populations was that the NTSR transcriptome contained a much greater number of contigs (290 946) than the 119 240 determined in the HS plants (Table 1). After normalizing relative abundance, 4724 Unigenes varied more than two‐fold between the HS and NTSR populations (*P* < 0.05, FDR < 0.05). Of these, 2908 sequences were matched to genes identified in the Uniprot database. Compared with HS plants, NTSR blackgrass had 1537 upregulated and 1371 downregulated transcribed genes. Using the Mercator annotation tool (Lohse *et al*., 2014), genes were assigned specific functions (Bin codes) and the contigs clustered into functional classes for comparison in NTSR and HS plants (Table 1). After applying this analysis, the range of gene classes with perturbed expression in the two populations was much reduced. With respect to primary metabolism, while the genes encoding photosynthetic components were unaffected, the NTSR plants had a lower proportion of transcripts associated with pathways linked to carbohydrate metabolism, glycolysis and fermentation. Similarly, many primary anabolic pathways leading to lipid, cell wall, nucleic acid and amino acid biosynthesis appeared to be suppressed in NTSR relative to HS plants.

**Table 1:** Whole transcriptomics of NTSR and HS blackgrass shoot tissue. Number of transcripts (contiguous sequences) in each functional category (BIN) is indicated for up and down regulation. (a) Mapping onto the NTSR or HS transcriptome assembly; (b) % of upregulated contigs relative to the number mapped onto the transcriptome; (c) fold changes across all functional categories following normalization

BIN code	BIN name	(a) Contig expression		(b) Contig mapping			(c) Expression versus mapping	
		Upregulated in NTSR	Upregulated in HS	All NTSR	All HS	% mapped in NTSR versus HS	% NTSR up‐reg contigs (# NTSR mapped contigs)	% HS up‐reg contigs (# HS mapped contigs)
1	Photosynthesis	739	143	15 164	2978	83.6	4.9	4.8
2	Major CHO metabolism	7	23	650	252	72.1	1.1	9.1
3	Minor CHO metabolism	10	49	718	328	68.6	1.4	14.9
4	Glycolysis	13	10	979	298	76.7	1.3	3.4
5	Fermentation	1	3	90	42	68.2	1.1	7.1
6	Gluconeogenesis	2	0	100	39	71.9	2.0	0.0
7	OPP	3	3	288	92	75.8	1.0	3.3
8	TCA/org. transformation	29	10	813	287	73.9	3.6	3.5
9	Mitochondrial electron transport/ATP synthesis	5	3	294	116	71.7	1.7	2.6
10	Cell wall	12	37	936	455	67.3	1.3	8.1
11	Lipid metabolism	33	30	1485	663	69.1	2.2	4.5
12	N‐metabolism	21	3	734	168	81.4	2.9	1.8
13	Amino acid metabolism	61	37	2121	723	74.6	2.9	5.1
14	S‐assimilation	8	0	118	25	82.5	6.8	0.0
15	Metal handling	13	5	206	74	73.6	6.3	6.8
16	Secondary metabolism	46	17	1189	446	72.7	3.9	3.8
17	Hormone metabolism	33	42	867	482	64.3	3.8	8.7
18	Cofactor and vitamin metabolism	14	9	425	170	71.4	3.3	5.3
19	Tetrapyrrole synthesis	40	11	997	204	83.0	4.0	5.4
20	Stress	27	40	1193	595	66.7	2.3	6.7
21	Redox	24	25	1084	377	74.2	2.2	6.6
22	Polyamine metabolism	1	3	69	44	61.1	1.4	6.8
23	Nucleotide metabolism	5	17	481	251	65.7	1.0	6.8
24	Biodegradation of xenobiotics	1	0	91	34	72.8	1.1	0.0
25	C1‐metabolism	40	5	624	122	83.6	6.4	4.1
26	Miscellaneous	114	78	1926	937	67.3	5.9	8.3
27	RNA	22	177	3101	1943	61.5	0.7	9.1
28	DNA	3	27	749	413	64.5	0.4	6.5
29	Protein	81	270	11 139	5043	68.8	0.7	5.4
30	Signalling	33	152	2438	1543	61.2	1.4	9.9
31	Cell	20	67	2193	1132	66.0	0.9	5.9
32	‘micro RNA, natural antisense etc.’	0	0	1	0	100.0	0.0	n/a
33	Development	18	39	1036	631	62.1	1.7	6.2
34	Transport	58	84	3343	1517	68.8	1.7	5.5
35	Not assigned	176	246	232 305	82 444	73.8	0.1	0.3
			Total:	290 946	119 240			

The genes associated with metabolism that were profoundly upregulated in NTSR plants (i.e. when the relative abundance in HS = 0) were restricted; namely those linked to sulphur assimilation, gluconeogenesis and xenobiotic detoxification. The fact that this reductionist approach to examining the herbicide resistance transcriptome identified this small cluster of biochemical functions was interesting. Of the three pathways identified, previous biochemical studies had identified both xenobiotic detoxifying enzymes and sulphur assimilation, in the form of glutathione synthesis, as being among the few clearly upregulated aspects of metabolism in NTSR blackgrass (Cummins *et al*., 1999, 2013). While these informatics approaches provided insight into the regulation and scale of NTSR at the level of gene expression, they provided little new understanding into the mechanisms associated with resistance. For this reason, an untargeted proteomics study was performed on NTSR versus HS plants to look for proteins that might be functionally correlated to this complex trait.

### Differential proteomics of NTSR versus HS blackgrass

**Figure 1:**
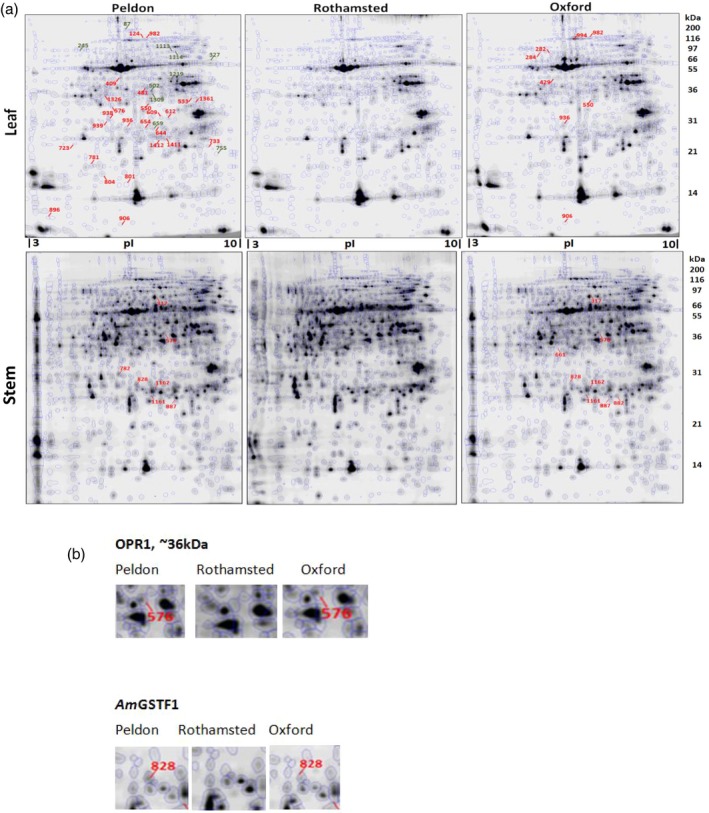
Proteome maps for leaf and stem tissue of NTSR (Peldon, Oxford) and HS (Rothamsted) blackgrass populations. (a) Polypeptide spots that were significantly enhanced (+1.5‐fold) in the NTSR populations compared to the HS are numbered and indicated on the maps. (b) Two representative protein spots illustrating their increased synthesis in the two NTSR populations.

As a first step in investigating the resistance proteome, total soluble protein extracts from HS Rothamsted and NTSR Peldon and Oxford populations were separated on large format two‐dimensional electrophoresis and differentially quantified using fluorescent dye protein labelling (DiGE). For comparison, leaves and stems were separately analysed to test the assumption that NTSR toward herbicides acting on photosystems and on meristems may be differentially presented in the two tissues. In total, 1037 protein spots were detected in stem tissue and 894 in the leaves, with the latter dominated by the Rubisco large (60 kDa) and small (14 kDa) polypeptide subunits (Figure 1). In both tissues, protein spots showing a significant (*P* < 0.05, fold change >1.5) difference in abundance were picked from the gels, subjected to controlled proteolysis and the polypeptides identified using a combination of MALDI‐TOF peptide mass finger‐printing and LC‐MS sequencing. Resulting spectra were matched to a translated sequence database assembled from the blackgrass transcriptome study, and subsequently blasted against available datasets from *Lolium*,* Brachypodium*, and rice in the NCBI database. All of the differentially abundant proteins identified by DiGE analysis are shown in Dataset S1. The gel images, protein quantification and mass spectrometry data have been deposited to the ProteomeXchange with identifier <PXD008759>.

**Figure 2:**
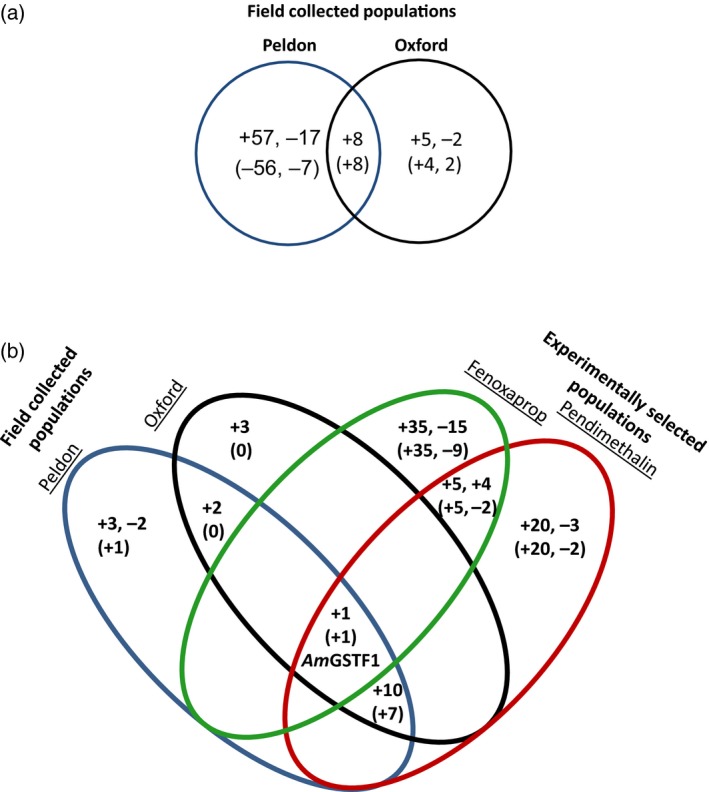
Number of (a) leaf and (b) stem polypeptide spots differentially changed in their abundance [increased (+) or reduced (−)] in NTSR populations compared with HS plants. This includes polypeptides that were differentially abundant in HS plants exposed to biotic and abiotic stress. Numbers in brackets denote polypeptides that were not differentially expressed under HS stress conditions. Only one protein (*Am*GSTF1) was found to be differentially enhanced in all four NTSR populations in stem tissue.

On comparison of the leaf proteomes, 89 polypeptide spots showed between a 1.5‐ to 15‐fold change in abundance in the two NTSR populations as compared with the HS plants (Figure 2(a)). Of these, a total of 62 polypeptide spots showed an increased abundance in the resistant plants, with 57 selectively enhanced only in Peldon and five exclusively in the Oxford plants. In the stem tissue, 77 polypeptide spots were enhanced and 22 repressed in the NTSR versus HS plants (Figure 2(b)). In the Peldon and Oxford plants, while the population‐specific perturbations in polypeptide abundance could reflect specific resistance protein expression, another plausible explanation was the inherent natural variation in the proteomes of these diverse blackgrass populations.

As such, interest was focused on the eight leaf and 17 stem polypeptides that were commonly enhanced in both Peldon and Oxford relative to HS plants (Figure 2(a,b)). The comparative analysis was concentrated on the stems due to the lower prevalence of the Rubisco‐linked polypeptides that dominated the leaf proteome, together with the greater relevance of this tissue hosting the pathways targeted by the ALS‐ and ACCase‐active herbicides used in this study. The identities of the sequenced polypeptides consistently linked to NTSR in the stems are summarized in Table 2. The same proteins also showed a common enhancement in the leaf tissue from both NTSR populations. Of the 17 polypeptides enhanced in the stems of both Peldon and Oxford populations (Dataset S1), 16 were subsequently sequenced and identified as being derived from just eight proteins. Of these proteins, the phi (F) class glutathione transferase, *Am*GSTF1, had previously been identified as being associated with NTSR in blackgrass (Cummins *et al*., 1999, 2013). *Am*GSTF1 is encoded by at least four gene sequences (Cummins *et al*., 1999), but in the current study, polypeptides from only two isoforms, namely the 2d (S106), and 2c (S125) variants were identified (Table 2 and Dataset S1). In addition to *Am*GSTF1, further GSTs from the phi (*Am*GSTF2; S105) and tau (U) (*Am*GSTU2, S129) classes were also determined. As detailed in Table 2, the other commonly enhanced polypeptides in the NTSR versus HS proteome were a 12‐oxophytodienoate reductase 1 (OPR1; S55), two isoenzymes of D‐3‐phosphoglycerate dehydrogenase 1 (PHGDH1 & 2; S25 & S27), a stem‐specific protein TSJT1 (S101), an NADPH:quinone oxidoreductase 1 (S103) and a NAD‐dependent epimerase/dehydratase (S102) isoenzyme (Table 2).

**Table 2:** Abundance of eight candidate NTSR‐associated proteins consistently determined in stem tissue and associated with NTSR and herbicide group‐specific resistance. Significant differences in fold‐abundance (*P* < 0.05, fold change >1.5) were relative to equivalent HS plants, with red being enhanced and green suppressed. For reference, differences in transcript abundances (NTSR versus HS) are also shown along with literature citing similar changes in protein expression associated with resistance to chemicals

Accession number	Protein spot number	Protein identification	Protein fold change in NTSR and HS stressed plants compared to HS control plants												Transcript fold change in NTSR compared to HS	Protein induction in other studies
			NTSR populations				Stress conditions applied to HS plants									
			Peldon	Oxford	Fenoxaprop	Pendimethalin	Wound	Rhizobacteria	Aphid	Heat	Drought	Salt	10% Nitrogen	Safener	Peldon	
KY172654	S101	Stem‐specific protein TSJT1	3.1	4.0	1.1	1.7	0.6	1.1	0.9	0.9	0.6	0.9	0.8	1.2	4.2	
KY172653	S55	12‐oxophytodienoate reductase 1	2.6	2.5	1.1	4.1	1.0	0.5	1.0	0.3	0.5	0.7	0.9	0.6	101.2	Herbicide (1)
KY172658	S27	D‐3‐phosphoglycerate dehydrogenase 1	4.0	3.4	1.2	3.2	1.4	1.0	1.3	0.2	1.1	1.2	1.2	1.3	5.3	Tumour (2)
KY172659	S105	GSTF2	**3.7**	3.5	1.1	2.2	–	–	–	–	–	–	–	–	6.1	
KY172655	S129	GSTU2	6.2	2.2	1.3	2.0	–	–	–	–	–	–	–	–	141.5	
KY172656	S102	NAD‐dependent epimerase/dehydratase	3.1	4.0	1.1	1.7	0.6	1.1	0.9	0.9	0.6	0.9	0.8	1.2	ns	
KY172657	S103	NADPH:quinone oxidoreductase 1	3.1	4.0	1.1	1.7	0.6	1.1	0.9	0.9	0.6	0.9	0.8	1.2	46.7	Herbicide (1); Tumour cells (3)
AJ010454.1	S106	AmGSTF1 (2d)	2.9	2.5	1.6	2.1	–	–	–	–	–	–	–	–	27.2	Tumour cells (4)

References: 1, Holmes *et al*. (2006); 2, Possemato *et al*. (2011); 3, Siegel *et al*. (2012); 4, Geng *et al*. (2013)

An obvious difficulty in identifying proteins causatively associated with resistance in different blackgrass populations was the natural genetic diversity in plants isolated from the field. To further refine the search for NTSR‐specific proteins and to reduce the interference of natural variation in the expression of proteins in different blackgrass populations, the proteomes of Rothamsted plants that had undergone repeated selection for resistance to fenoxaprop, or pendimethalin, were then compared with that of the Peldon, Oxford and HS plants. As determined with the field populations, effort was focused on the comparative proteomics of the stem tissue. In total, 1100 discreet polypeptides were identified in these studies, with 69 differentially abundant when NTSR plants were compared with their HS counterparts (Dataset S1). On comparing those polypeptides with greater abundance following selection with fenoxaprop, or pendimethalin, with the field NTSR populations, only *Am*GSTF1 was consistently enhanced in all resistant plants (Figure 2(b)).

The results with the two ‘forced‐resistance’ populations showed surprising differences both between each other and with the field‐selected populations. The pendimethalin‐selected line, that showed NTSR toward all three ACCase inhibitors (fenoxaprop, clodinafop, and cycloxydim), but not toward the herbicides targeting ALS or cell division (Table S3), shared a common 10 upregulated protein spots with those also enhanced in the field collected NTSR populations. Of these 10, the most similarly upregulated in the Peldon and Oxford plants were *Am*GSTF1, *Am*GSTF2, *Am*GSTU2, OPR1 and PHGDH 1 (Table 2 and Dataset S1). In contrast, the fenoxaprop‐selected line, that only showed NTSR toward this one ACCase inhibitor, had a distinct proteome profile from both the field‐derived and pendimethalin‐resistant populations (Table 2 and Dataset S1). The proteins upregulated in the fenoxaprop line included *O*‐acetylserine (thiol)‐lyase (functioning in cysteine synthesis; S283), UDP‐glycosyltransferase (UGT91C1; secondary metabolism; S282), eukaryotic translation initiation factor 3 subunit I (hormone metabolism; S64), uroporphyrinogen decarboxylase 1 (tetrapyrrole biosynthesis; S271), and triosephosphate isomerase (energy and metabolism; S110).

### Comparative proteomics of NTSR and stress responses in blackgrass

Having established that a small group of proteins were consistently associated with NTSR in blackgrass, it was then of interest to compare this proteome resistance signature with that determined when the plants were exposed to common stress treatments. Rothamsted (HS) blackgrass plants were exposed to long‐term abiotic stress treatments (nitrogen deficiency, and prolonged exposure to salt and drought) as well as more transient challenges (wounding and heat shock). The plants were also exposed for 14 days to biotic stress (aphid feeding and inoculation with rhizobacteria). Finally, the plants were exposed to herbicide safeners.

In each case, after the treatment and at the time of proteome sampling, the plants were assessed for changes in root and shoot biomass (Figure S1(a)). This experiment demonstrated that the blackgrass plants showed typical responses to environmental challenge, with a marked reduction in aerial biomass observed with the long‐term stress treatments and increased root growth elicited by N‐deprivation. In the field‐derived NTSR plants no overt differences in root and shoot growth were observed between HS and NTSR plants (Figure S1(b)). In contrast, the Rothamsted plants that had undergone repeated herbicide selection for resistance to fenoxaprop did show a reduced growth in both roots and shoots suggestive of a fitness penalty in that class of NTSR (Figure S1(c)).

**Figure 3:**
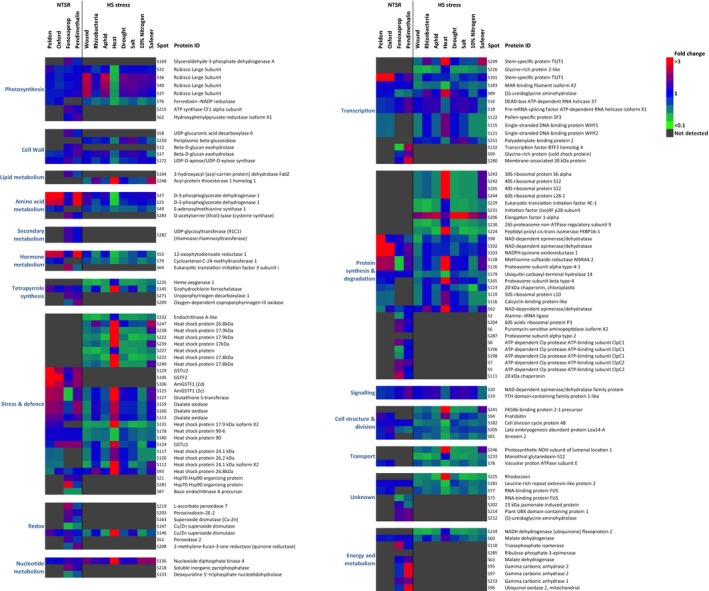
Functional categorization and abundance of NTSR and HS stress response proteins detected in stem tissue relative to HS control plants. Proteins not detected in a population or stress condition are shaded black; blue represents no differential abundance, red and green represent increased or decreased abundance respectively. This finding indicates that protein spots with increased abundance in NTSR populations were either not differentially enhanced, or were depressed in the HS plants exposed to stress treatments.

All the biotic and abiotic stress treatments elicited changes in the blackgrass proteome (Dataset S1). However, in all cases, the changes determined in these imposed stress treatments had little in common with the constitutive NTSR protein expression phenotype (Table 2 and Figure 3). Around 18 polypeptides identified in leaf and stem tissue associated with NTSR were also more abundant in heat stressed plants (Dataset S1 and Figure 3). These included photosynthesis‐related proteins, enzymes linked to detoxification, *Am*GSTF1(2c) and *Am*GSTU2, pathogen defence oxalate oxidase, tetrapyrrole synthesis (sirohydrochlorin ferrochelatase) and a superoxide dismutase (redox). However, heat stress also increased of proteins that were depressed in the NTSR proteome, demonstrating there were no consistent functional links between the two responses.

To broaden the comparative analysis to other species, the genes most strongly upregulated by NTSR in the blackgrass transcriptome study were examined in Arabidopsis and rice exposed to a variety of stresses using Genevestigator (Figure S2). Using the top 11 NTSR upregulated genes identified in the whole transcriptomics data from blackgrass it was clear using a ‘heat map induction’ comparison that there was no consistent stress induction of the orthologous genes examined in any of the species analysed (Figure S2).

One of the most obvious comparisons within this study was to determine any similarities in changes in the blackgrass proteome following safener‐treatment with that determined in NTSR plants. To test this, on exposure of HS blackgrass to the wheat safener cloquintocet‐mexyl, major changes in the proteome were determined, with 25% of the resolved polypeptides in leaves (131 of 512 protein spots), and 3% of the resolved stem proteins (26 of 874 protein spots) showing changes in abundance relative to controls. Only a minority of polypeptides that showed perturbation in NTSR underwent similar changes when the HS plants were exposed to the safener. One exception was *Am*GSTF1 (the 2c isoform), that was enhanced following safener‐treatment and was constitutively upregulated in all the NTSR populations showing cross‐resistance to multiple herbicides (Table 2). With respect to polypeptides showing a common suppression in NTSR and safening, a group of stem proteins sequenced from a single protein spot containing a mixture of heat shock proteins, a ribosome component, a ubiquitin degradation factor and transcriptional regulator were all repressed. However, the majority of NTSR‐associated polypeptides were either not detected in the safener‐exposed blackgrass, or found to undergo diametrically opposed changes in their relative abundance (Dataset S1 and Figure S3).

While the effects of safening on the blackgrass proteome did not resemble NTSR, several similarities were observed in safener‐treated plants with those exposed to the stress treatments. Of the 132 safener‐induced polypeptides resolved in the leaf tissue, 129 were also similarly differentially synthesized in response to at least one of the biotic/abiotic treatments, with the greatest similarity seen following nitrogen deficiency (Figure 3 and Dataset S1). In the stems, 25 out of 26 of the safener‐induced changes in polypeptide abundance were also elicited by stress conditions, mostly notably heat treatment (Figure S3). However, a further 148 polypeptides in the stems were enhanced by stress conditions but by the safener. Overall the results demonstrated that while there was overlap in the changes in protein expression elicited by safener with those seen following biotic/abiotic stress, there were no obvious functional links to the NTSR phenotype.

## Discussion

As determined in other wild grasses (Peng *et al*., 2010; Riggins *et al*., 2010; Yang *et al*., 2013; Gaines *et al*., 2014; Gardin *et al*., 2015), NTSR in different blackgrass field populations in our study was associated with the perturbed expression of over 4000 genes as compared with HS plants. In contrast, only a small number of soluble proteins showed any significant (>1.5‐fold) perturbation associated with this type of resistance. This 1.5‐fold threshold was chosen to reflect changes in protein abundance that were more likely to be of functional significance as defined from earlier studies on changes in GST content associated with herbicide resistance in this weed (Cummins *et al*., 1999, 2013). An important caveat to this observation was that the current study did not examine changes in the expression of membrane‐bound polypeptides and would therefore likely under‐estimate global changes in protein expression. This is particularly significant when factoring in the known important roles of membrane associated CYPs and transporter proteins in NTSR (Yuan *et al*., 2007; Busi *et al*., 2013; Gaines *et al*., 2014; Délye *et al*., 2015). However, in terms of exemplifying the disconnect between transcription and proteome composition, of the 67 identified proteins in Peldon showing an enhanced abundance relative to HS plants, only 29% showed any induction of the respective genes.

While natural genetic variation between populations might account for some of the major changes in transcription observed, a striking difference in the Peldon versus Rothamsted blackgrass was that the NTSR plants contained twice as many transcripts in total compared with the HS population. This finding suggests that NTSR is correlated with global changes in gene expression, which are then attenuated through the mass turnover of transcripts and/or translated proteins, such that the changes in steady state protein expression are modest. The changes in transcription associated with NTSR may reflect an acquired greater plasticity in gene expression resulting from environmental stress, allowing for a more rapid evolution of resistance to herbicides. Previously, we had speculated that increased transcription may result from infection of blackgrass by cryptic viruses, with many NTSR populations showing such infection (Sabbadin *et al*., 2017). Surprisingly, as determined by the simple biomass studies (Figures S1 and S2), these large‐scale increases in transcription and their associated metabolic cost provoked no obvious fitness penalty for NTSR at the whole plant level.

The ability to focus on the small number of proteins consistently associated with NTSR and to then study their variation in content in the different resistant populations, provided new insight into the potential mechanisms of this type of herbicide resistance. Effectively three types of NTSR could be identified from the field and forced‐selection studies with each associated with changes in the respective proteomes. Firstly, a comprehensive NTSR was identified in the field‐derived Peldon and Oxford populations that was associated with multiple resistance to both ALS and ACCase‐active herbicides. We have previously termed this multiple‐herbicide resistance (MHR), as it is independent of herbicide chemistry and mode of action (Cummins *et al*., 2013). Then there was the group‐specific class of NTSR determined in the pendimethalin‐selected plants that only conferred cross‐resistance to different ACCase‐active herbicides. Finally, we determined a compound‐specific type of NTSR that conferred resistance to only a single herbicide, as selected by fenoxaprop.

These experiments clearly demonstrated that NTSR in grass weeds can take multiple forms following different imposed selection regimes. None of the ‘constitutive’ proteomes of the NTSR‐types resembled those of any of the biotic and abiotic treatments applied. This observation is particularly pertinent to the effects of safener‐exposure. It has been postulated that the continuous exposure of grass weeds to the safeners used in many post‐emergence herbicide formulations could pre‐dispose them to becoming more herbicide tolerant through an NTSR‐related mechanism, presumably though an epigenetic route (Délye *et al*., 2013). Our previous studies have demonstrated that exposure of HS blackgrass to safeners does indeed give a minor enhancement to the rate of herbicide detoxification (Cummins *et al*., 2009). Furthermore, recent studies have reported that safeners can enhance herbicide tolerance in *Lolium* sp. (Duhoux *et al*., 2017). However, the current proteomics study suggests that this weak safening in grass weeds is unlikely to be functionally linked to the evolution of NTSR.

The three different types of NTSR (MHR, group & compound specific), were each associated with characteristic changes in their proteomes. In terms of identifying those proteins associated with the different types of NTSR, as shown in Table 2, only *Am*GSTF1 was found to be upregulated in all three classes of resistance. This further confirmed the importance of this protein as a core component of NTSR in blackgrass (Cummins *et al*., 1999, 2013). Similarly, orthologues of *Am*GSTF1 have also been shown to be upregulated in NTSR in other wild grasses, such as *L*. *rigidum* (Cummins *et al*., 2013). While the fenoxaprop‐specific NTSR clearly exemplified the potential for unique evolutionary routes to NTSR, the changes in the respective proteome gave no immediate clues as to the resistance mechanisms in play.

In contrast, the proteins upregulated in both the group‐specific and MHR blackgrass did give some insight into NTSR mechanisms. Four of the eight proteins, identified were xenome components, suggesting a strong functional link of NTSR to detoxification. In addition to *Am*GSTF1, a further phi‐class protein termed *Am*GSTF2 and a tau enzyme *Am*GSTU2 were identified. The latter was of interest, as it was by far the most abundant GST in the MHR plants. To date, it has not proven possible to predict plant GST function from sequence alone, but the elevation of a group of these proteins in NTSR plants, linked to a coordinated elevation in glutathione content (Cummins *et al*., 2013), suggests they have complimentary functions in protecting the plants from herbicide injury. Another xenome protein of interest was OPR1. The OPRs are flavin mononucleotide‐binding enzymes that are classically associated with the conversion of 12‐oxo‐*cis*‐10,15‐phytodienoate to 3‐oxo(‐*cis*‐2′‐pentenyl) cyclopentane‐1‐octanoate, a key step in jasmonic acid biosynthesis (Stintzi and Browse, 2000). The OPRs can be subdivided into three classes, with the OPR1 enhanced in the NTSR plants, associated with the reductive detoxification of xenobiotics such as the explosive TNT, rather than jasmonate synthesis (Beynon *et al*., 2009).

The other polypeptides associated with MHR and group‐specific NTSR had not previously been linked to herbicide resistance in plants, but have been linked to multiple drug resistance (MDR) in human tumour cells. This was particularly interesting given the many similarities in enzyme chemistry previously determined between *Am*GSTF1 and its evolutionarily distant, but functionally orthologous human Pi‐class *Hs*GSTP1 that is closely linked to MDR in cancerous cells (Tew *et al*., 2011; Cummins *et al*., 2013). Now in addition to the *Am*GSTF1, a further two proteins were identified in NTSR (MHR and group‐specific) that were linked to MDR. The orthologue of the NADPH‐dependent quinone oxidoreductase (NQO1) in humans is over‐expressed in glioblastomas along with *Hs*GSTP1 (Okamura *et al*., 2000). The role of NQO1 in multi‐drug resistance is complex, as in addition to reducing quinone drugs to the more reactive hydroquinone, its catalytic mechanism also allows the protein to be a potent protective superoxide scavenger (Siegel *et al*., 2012). A further link between MHR in blackgrass and MDR in humans was the enhanced expression of D‐3‐phosphoglycerate dehydrogenase (PHGDH). This enzyme catalyzes the first committed step in the non‐photosynthetic (phosphorylated) pathway leading to l‐serine and ultimately cysteine and glycine in both plants and animals. As plants were thought to synthesize their l‐serine largely from the distinct photosynthetic pathway, relatively little is known about the role of PHGDH in metabolism, though it has recently been implicated in developmental signalling *(*Toujani *et al*., 2013). Interestingly, by mechanisms that are yet to be defined, the elevated expression of the PHGDH orthologue in humans is associated with multiple drug resistance and associated cell re‐differentiation in tumour cells (Possemato *et al*., 2011). From these proteomic studies, we therefore conclude that there is more to functionally link NTSR to MDR in humans, than to any response to abiotic or biotic stress in plants. Further studies will now focus on the mechanisms by which these NTSR‐associated proteins confer resistance to xenobiotics more widely in eukaryotes.

## Experimental procedures

### Plants and treatments

The herbicide resistance phenotypes of the HS (Rothamsted) and the field‐derived NTSR blackgrass populations Peldon and Oxford were as previously characterized (Hall *et al*., 1995; Moss *et al*., 2003). The NTSR selection lines derived from repeated application of either fenoxaprop or pendimethalin in controlled studies were derived from a Rothamsted population harvested in 1990. The repeated selections (Table S1). Subsequent herbicide testing procedures (Table S2) and resulting resistance profile testing (Table S3) were conducted at Rothamsted Research using the scoring system described (Stephen *et al*., 2007). For the transcriptome and proteome studies, blackgrass plants were grown and maintained as described previously (Cummins *et al*., 2013). For transcriptome work, seedlings were used at 21 days, while for proteome studies plants were extracted at 38 days (including stress treatments). The stress treatments were carried out as detailed in Methods S1.

### Proteomics

Proteins were extracted from separately from leaf and stem tissue (600 mg) and then prepared by trichloroacetic acid/acetone precipitation, purified and quantified as described previously (Tétard‐Jones *et al*., 2013). Each protein sample was prepared from eight plants grown within the same pot, with three biological replicates for each resistant population and stress treatment examined. Protein samples (50 μg) were labelled with minimal CyDyes, including an internal standard composed of an aliquot of each sample. Resulting scanned gel images were analysed using Progenesis SameSpots software (Non‐Linear Dynamics). Overall, 800–1100 distinct protein spots were matched across the DiGE gels. The normalized protein spot volume (a measure of spot area and intensity) was analysed between each stress treatment and NTSR population with their respective HS control, using anova (nlme package in R) as described (Team, 2013), to ascertain significant differences in protein abundance. The false discovery rate (FDR) was determined using published methodology (Benjamini and Hochberg, 1995), using 10% as the acceptable FDR for proteomics data (Artigaud *et al*., 2013). Protein spots that were significantly differentially abundant between blackgrass populations and stress treatments as compared to their respective controls (*P* < 0.05 and fold change >1.5), were selected for protein identification using a combination of MALDI‐TOF peptide mass finger‐printing and LC‐MS sequencing (Bioscience Technology Facility, University of York). Protein spots were manually picked from Coomassie stained ‘preparative’ gels as described previously (Tétard‐Jones *et al*., 2013). Peptide spectra were matched to peptide sequences against a bespoke blackgrass translated transcriptome database using Mascot (2016), and blasted against known proteins in NCBI. The search parameters for matching spectra were: fixed modification carbamidomethyl (C), variable modifications deamidated (NQ) and oxidation (M), peptide mass tolerance ± 100 ppm, fragment mass tolerance ±0.5 Da and maximum missed cleavages 1. The blackgrass translated database consisted of 2 241 330 sequences (708 477 927 residues). This compared with the NCBI database, consisting of 73 792 625 sequences (26 861 771 399 residues). In each case, the top match in the NCBI database was reported as detailed (Table 2 and Dataset S1). All matches had an identity score of >90%, and E (Expect) value <0.0. The identity score representing the extent to which two sequences have the same residues at the same position in an alignment, while the E value represents the number of different alignments with scores equivalent to, or better than, S that is expected to occur in a database by chance. The mass spectrometry proteomics data have been deposited to the ProteomeXchange Consortium (http://proteomecentral.proteomexchange.org) via the MassIVE partner repository with the dataset identifier <PXD008759>.

## Acknowledgements

The authors acknowledge joint grant funding from the Biotechnology and Biological Sciences Research Council (BB/L001489/1) and the Agriculture and Horticulture Development Board. The authors wish to thank the Metabolomics & Proteomics laboratory at the Bioscience Technology Facility, University of York for analysing samples.

## Conflict of interest

The authors declare no conflict of interest.

## Supporting information


**Figure S1.** Fresh biomass of (a) Rothamsted (HS) plants following exposure to a range of biotic and abiotic stress treatments; (b) field‐sourced NTSR plants; and (c) experimentally selected NTSR plants.
**Figure S2.** Stress inducibility of orthologous genes in (a) *Arabidopsis thaliana*; and (b) *Oryza sativa* corresponding to the top 10 upregulated NTSR blackgrass transcripts and candidate protein biomarkers.
**Figure S3**. Percentage similarity of differentially abundant leaf protein spots in paired comparisons between stress treatments and NTSR populations.

**Table S1**. Treatment programme used to experimentally select for fenoxaprop resistance in blackgrass grown in outdoor containers, starting with the Rothamsted HS population in 1990.
**Table S2.** Herbicide resistance testing regime used with a range of graminicides on blackgrass repeatedly selected for using pendimethalin (PEND) and fenoxaprop (FEN).
**Table S3.** Cross‐resistance of experimentally selected herbicide‐resistant populations.

**Dataset S1.** Differential abundance and characterisation of leaf (a) and stem (b) proteins across NTSR populations and HS stress treatments compared with the HS control.

**Methods S1.** Supplementary materials.
