# Supplementary material for: Changes in the proteome of the problem weed blackgrass correlating with multiple‐herbicide resistance
Source: Plant J. 2018 Apr 25;94(4):709–20. doi: 10.1111/tpj.13892 (PMC5969246; doi:10.1111/tpj.13892)
Supplement: Supplementary file 1 — Figure S1. Fresh biomass of (a) Rothamsted (HS) plants following exposure to a range of biotic and abiotic stress treatments; (b) field‐sourced NTSR plants; and (c) experimentally selected NTSR plants. Figure S2. Stress inducibility of orthologous genes in (a) Arabidopsis thaliana; and (b) Oryza sativa corresponding to the top 10 upregulated NTSR blackgrass transcripts and candidate protein biomarkers. Figure S3. Percentage similarity of differentially abundant leaf protein spots in paired comparisons between stress treatments and NTSR populations. [file TPJ-94-709-s001.docx]

**SUPPORTING INFORMATION**

**Figure S1.** Fresh biomass of A) Rothamsted (HS) plants following exposure to a range of biotic and abiotic stress treatments, B) Field sourced NTSR plants, C) experimentally selected NTSR plants. All plants were 38 days old when harvested.





**Figure S2.** Stress inducibility of orthologous genes in A) *Arabidopsis thaliana*, B) *Oryza sativa* corresponding to the top ten up-regulated NTSR blackgrass transcripts and candidate protein biomarkers. Colours relate to the up-regulation (red), neutral (yellow) or down-regulation (green) of genes.


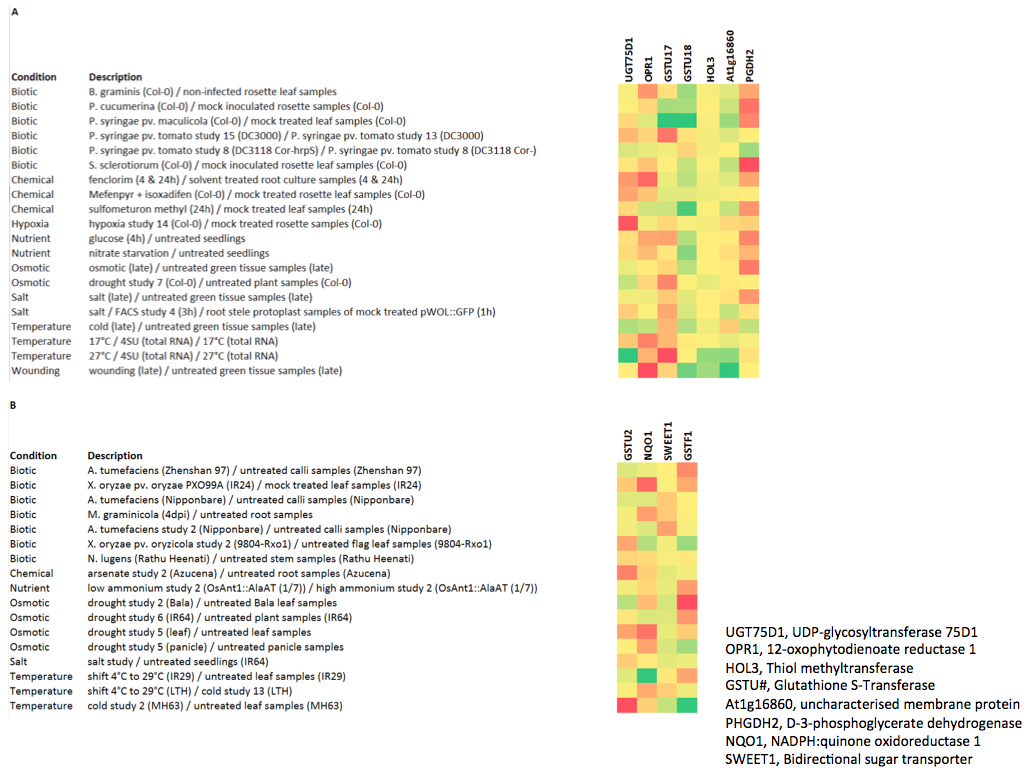


**Figure S3**. Percentage similarity of differentially abundant leaf protein spots in paired comparisons between stress treatments and NTSR populations. The intensity of colours is related to the degree of similarity between paired comparisons.
