# Supplementary material for: Changes in the proteome of the problem weed blackgrass correlating with multiple‐herbicide resistance
Source: Plant J. 2018 Apr 25;94(4):709–20. doi: 10.1111/tpj.13892 (PMC5969246; doi:10.1111/tpj.13892)
Supplement: Supplementary file 2 — Table S1. Treatment programme used to experimentally select for fenoxaprop resistance in blackgrass grown in outdoor containers, starting with the Rothamsted HS population in 1990. Table S2. Herbicide resistance testing regime used with a range of graminicides on blackgrass repeatedly selected for using pendimethalin (PEND) and fenoxaprop (FEN). Table S3. Cross‐resistance of experimentally selected herbicide‐resistant populations. [file TPJ-94-709-s002.docx]

**Table S1.** Treatment program used to experimentally select for fenoxaprop resistance in blackgrass grown in outdoor containers, starting with the Rothamsted HS population in 1990. Variations in the doses used for each round of selection reflect changes in yearly environmental conditions.

| Selection year | Year | Nos. plants treated | Nos. plants grown on for seed production | Selection  % | Selecting dose |
| --- | --- | --- | --- | --- | --- |
| 1^st^ | 1990 | 456 | 10 | 2.2% | 70 g fenoxaprop racemate/ha  (= x 0.58 field rate) |
| 2^nd^ | 1991 | 506 | 3 | 0.6% | 100+100 g fenoxaprop racemate/ha (= x 1.67 field rate) |
| 3^rd^ | 1991/92 | 524 | 15 | 2.9% | 125 g fenoxaprop racemate/ha  (= x 1.04 field rate) |
| 4^th^ | 1997/98 | 486 | 43 | 8.8% | 27.5 g fenoxaprop isomer/ha  (x 0.5 field rate) |
| 5^th^ | 1998/99 | 50 | 8 | 16% | 220 g fenoxaprop isomer/ha  (x 4 field rate) |
| 6^th^ | 2014/15 | 26 | 26 | 100%  All survived | 18 plants x 55 g fenoxaprop isomer/ha  + 8 plants x 110 g fenoxaprop isomer/ha |

**Table S2.** Herbicide resistance testing regime used with a range of graminicides on blackgrass repeatedly selected for using pendimethalin (PEND) and fenoxaprop (FEN). The HS Rothamsted populations they were selected from were also tested. Each treatment was carried out on 6 plants (n=3). Applications were either at full (#) or ¾ (*) field rate

**Populations (4**): HS ROTH04 (used to generate the PEND)

WTS ROTH09 (used to generate the FEN)

ROTH PEND (8yrs selection)

ROTH FEN (6yrs selection)

**Treatments (9):** 1 Untreated

2 Fenoxaprop* 0.75 l/ha

3 Clodinafop* 0.0938 l/ha + 0.5% vol. Toil

4 Cycloxydim* 0.563 l/ha + 0.5% vol. Toil

5 Mesosulfuron+iodosulfuron* 300 l/ha + 0.5% vol. BioPower

6 Sulfometuron* 99.75 g/ha

7 Pyroxsulam* 250 g/ha + 1.0% Biosyl

8 Pendimethalin# 1200g ai/ha

9 Flufenacet# 240g ai/ha

**Table S3.** Cross resistance of experimentally selected herbicide resistant populations.

Two populations originating from the HS population Rothamsted collected in 2004 (Roth04) selected for resistance against either Fenoxaprop (6-year selection) or Pendimethalin (8-year selection) demonstrated marked differences in their cross resistance to other herbicides. “Roth Pendimethalin” was resistant against all tested ACCase inhibitors, whereas “Roth Fenoxaprop” was only resistant against Fenoxaprop and susceptible to all other tested herbicides. Plants originating from the HS population Rothamsted collected in 2009 (Roth09) were included to show that resistance did not naturally occur over the selection period. *Abbreviations for herbicidal mode of action, ACCase = Acetyl CoA Carboxylase (lipid synthesis), ALS = Acetolactate (branched chain amino acid formation); ^$^ Resistance ratings follow the method of Moss et al. (8)

| Herbicide and mode of action* | Population | Dry weight tissue (DWT) | | Mortality | |  |
| --- | --- | --- | --- | --- | --- | --- |
|  |  | Reduction in DWT (%) | Resistance rating^$^ | Plant mortality (%) | Resistance rating^$^ |  |
| Fenoxaprop, ACCase inhibitor | Roth04 | 81.30 | S | 100.0 | S |  |
|  | Roth09 | 74.03 | S | 100.0 | S |  |
|  | Roth Pendimethalin | 39.33 | RR | 38.9 | RRR |  |
|  | Roth Fenoxaprop | 53.85 | RR | 50.0 | RRR |  |
| Clodinafop, ACCase inhibitor | Roth04 | 79.13 | S | 100.0 | S |  |
|  | Roth09 | 77.70 | S | 100.0 | S |  |
|  | Roth Pendimethalin | 33.23 | RR | 66.7 | RR |  |
|  | Roth Fenoxaprop | 84.29 | S | 100.0 | S |  |
| Cycloxydim, ACCase inhibitor | Roth04 | 74.80 | S | 100.0 | S |  |
|  | Roth09 | 76.16 | S | 100.0 | S |  |
|  | Roth Pendimethalin | 45.99 | RR | 83.3 | R |  |
|  | Roth Fenoxaprop | 91.62 | S | 100.0 | S |  |
| Mesosulfuron+Iodosulfuron, ALS inhibitor | Roth04 | 68.09 | S | 100.0 | S |  |
|  | Roth09 | 65.19 | S | 100.0 | S |  |
|  | Roth Pendimethalin | 77.31 | S | 100.0 | S |  |
|  | Roth Fenoxaprop | 83.24 | S | 100.0 | S |  |
| Sulfometuron, ALS inhibitor | Roth04 | 76.97 | S | 100.0 | S |  |
|  | Roth09 | 74.54 | S | 100.0 | S |  |
|  | Roth Pendimethalin | 78.32 | S | 100.0 | S |  |
|  | Roth Fenoxaprop | 85.71 | S | 100.0 | S |  |
| Pyroxsulam, ALS inhibitor | Roth04 | 74.96 | S | 100.0 | S |  |
|  | Roth09 | 68.06 | S | 100.0 | S |  |
|  | Roth Pendimethalin | 84.27 | S | 94.4 | S |  |
|  | Roth Fenoxaprop | 88.66 | S | 100.0 | S |  |
| Pendimethalin, microtubule assembly inhibition | Roth04 | 7.61 | * | 55.6 | S |  |
|  | Roth09 | 10.38 |  | 61.1 | S |  |
|  | Roth Pendimethalin | -4.99 |  | 5.6 | RRR |  |
|  | Roth Fenoxaprop | 36.60 |  | 88.9 | S |  |
| Flufenacet, cell division (very long chain fatty acids) inhibition | Roth04 | 78.34 | S | 100.0 | S |  |
|  | Roth09 | 53.57 | S | 88.9 | S |  |
|  | Roth Pendimethalin | 66.41 | S | 88.9 | S |  |
|  | Roth Fenoxaprop | 72.34 | S | 100.0 | S |  |
