# Supplementary material for: Changes in the proteome of the problem weed blackgrass correlating with multiple‐herbicide resistance
Source: Plant J. 2018 Apr 25;94(4):709–20. doi: 10.1111/tpj.13892 (PMC5969246; doi:10.1111/tpj.13892)
Supplement: Supplementary file 4 — Methods S1. Supplementary materials. [file TPJ-94-709-s004.docx]

**S1 Supplementary Materials and Methods for**

**Changes in the proteome of the problem weed blackgrass correlating with multiple-herbicide resistance**

Catherine Tétard-Jones^a^, Federico Sabbadin^b^, Stephen Moss^c^, Richard Hull^c^, Paul Neve^c^ & Robert Edwards^a*^,

School of Agriculture, Food and Rural Development, Newcastle University, Newcastle Upon-Tyne, NE1 7RU, UK.

^b^ Department of Biology, University of York, York YO10 5DD, UK.

^c^ Rothamsted Research, Harpenden, Hertfordshire AL5 2JQ.

*Corresponding author: Prof. Robert Edwards, School of Agriculture, Food and Rural Development, Newcastle University, Newcastle Upon-Tyne, NE1 7RU.

**Methods**

**Plant maintenance and stress treatments**

Black-grass were grown for 2.5 weeks (to 2 tiller stage) in compost (John Innes No.2) and then transferred to 10 cm pots (8 plants per pot, 3 pots per treatment and population) containing autoclaved sharp sand mixed with 25% perlite. Growth conditions were set to a light:dark cycle of 16:8 with temperature of 21^o^C:15^o^C and plants watered with full strength Hoagland’s solution. Plant tissues (leaf and stem) were harvested at 5.5 weeks old (6 tiller stage), weighed for fresh biomass, snap frozen in liquid nitrogen and stored at -80^o^C. To test whether NTSR associated constitutive proteins are stress inducible, we designed a multi-stress experiment consisting of eight stress treatments that black-grass could naturally be exposed to in the field. These consisted of i) biotic stresses: plant growth promoting rhizobacteria (PGPR), insect (aphid – *Sitobion avenae*) abiotic (wound, heat, drought, salt, nitrogen deficiency and a commonly used wheat safener (cloquintocet-mexyl). The stress conditions were applied at time-points consistent with their nature to ensure a common harvest point. Nitrogen deficiency (10% N Hoaglands recipe), salt stress (building from 40mM to 160mM NaCl diluted in Hoaglands) and rhizobacteria (10ml *Pseudomonas aeruginosa* 7NSK2 inoculum per plant, prepared as previously described (1), were applied three weeks before harvest. Two weeks before harvest, the insect stress (two adult “english grain” aphids, *Sitobion avenae*) were placed on each plant and insect proof bags fitted to all experimental plants. Aphids were allowed to naturally reproduce, reaching an average population of 281 individuals at harvest. The drought (osmotic) stress was artificially controlled, by application of Polyethylene Glycol (PEG) 8000 over 11 days prior to harvest, starting with an osmotic pressure of -0.15 MPa building up to -0.66 MPa as previously described (2). The safener (30mM cloquintocet-mexyl) was watered onto plants 4 days before harvest. The heat stress was applied as a gradual ramp from 21 ^o^C to 40 ^o^C in 3 ^o^C increments over 6 hours, and maintained at 40 ^o^C for a further 9 hours before harvest. The gradual heat ramp was intended to simulate a natural rising temperature, acclimating the plants to heat stress, to induce a heat response without protein degradation that can be caused by sudden heat shock (3). Wounding stress was applied by pressing a sewing wheel along the length of each plant leaf 9 hours prior to harvest.

**Transcriptomics**

NTSR (Peldon) and HS (Rothamsted) seed lines of black-grass were planted into 12 cm diameter terracotta pots in a peat based compost using 20 seeds per pot. Plants (3 biological replicates) were grown under controlled glasshouse conditions at the University of York and The Food and Environment Research Agency (Fera). After 3 weeks of growth plants were harvested directly into liquid nitrogen, cutting from just above the soil line and stored at -80 ^o^C for less than 2 weeks prior to transcriptomics analysis. We performed transcriptomics using IonTorrent next generation sequencing (see below)

**IonTorrent transcriptome sequencing and assembly**

RNA was extracted from frozen plant shoot tissue using RNeasy Mini Kit (Qiagen) according to the manufacturer`s protocols. Total RNA (15-20 ug) was DNase treated (Turbo DNA-free; Ambion) and then concentrated using the RNA Clean & Concentrate kit (Zymo Research). mRNA was purified from the total RNA using two passages through the Oligotex mRNA Mini kit (Qiagen) and quality profiles assessed on a 2200 TapeStation Nucleic Acids System (Agilent Technologies). RNA-Seq libraries were prepared from each mRNA sample (approx 25-50 ng) using the Ion Total RNA-Seq kit v2 (Life Technologies), with an RNase III treatment time of 2.5 - 3 min. Yields and library sizes were assessed using the 2200 TapeStation. Diluted library aliquots were combined in pairs in equimolar amounts and used for template preparation using the Ion OneTouch 200 Template Kit v2 DL (Life Technologies), prior to loading onto a 318 chip and sequencing on an Ion Torrent PGM prepared as per the manufacturer's instructions (IonPGM200Kit; Life Technologies).

*De novo* assembly of sequenced mRNA libraries was performed by Fios Genomics. Assembled BAM files were converted to SAM files and then into FASTQ format files using SamTools (4) and SamToFastq (v1.9; Picard tools). The FASTQC versions of the files were then assessed by Fastqc version 0.10.1 (FastQC) and reads trimmed to remove the first 10 bases at the 5’ end. The 3’ end of the reads were also trimmed wherever the quality scores dropped below 20. Any reads shorter than 40 nt were excluded from further analysis. rRNA and tRNA species from rice (*Oryza sativa*) were used as a proxy for the orthologs in black grass. These sequences were taken from the genome and gff files downloaded as part of IRSGP (build 4). Fasta sequences of the orthologs in the gff files were extracted using bedtools (5), the RNA-Seq reads were matched using bowtie2 using Ion-torrent specific settings: (local alignments, very-sensitive), as described by Langmead et al. (6). Those reads that did not match the rRNA species were considered in the later stages of the assessment.

*Assembly* – MIRA (7) was utilized for the assembly, which is considered to be the most reliable algorithm in instances where the assembly is generated from Ion-Torrent and not guided by a reference sequence, as in *de novo* transcriptome assembly (8, 9). The trimmed, rRNA filtered, read files from runs of the second replicate of either population were concatenated and used in two individual, accurate, EST specific assemblies. The reads that were left unassembled in either case and reads from the unused runs (first and third replicate of both populations) that failed to map to the generated contigs from the same run (using bowtie2 as previously described), were then combined. These additional collections of reads were used in two additional MIRA accurate EST assemblies. Reads from either assembly that had no further alignment were discarded.

*Generation of the Unigene sets* – The assembled contigs from both black-grass populations were combined and clustered into representative sequences using the CD-HIT-EST (10). All sequences with a gapped alignment of 95% over a minimum 90% of the shorter reads length were clustered. One set was generated for each population, and a final set was generated from the combined contigs of both populations.

*Annotation & super-contig assembly (scaffolding by association)* – Blast databases were generated from each of the following sources: NCBI’s species-specific unigenenucleic acid databanks for rice, barley (*Hordeum vulgare*) and *Arabidopsis thaliana* were used for KOG (coreprotein set), uniprot (protein) and finally the PlantCyc enzyme set analysis. The putative function of the contigs was assigned by blasting each sequence against the reference plant databases in turn, using either blastx for protein databases, or blastn for nucleic acid based databases. Only matches where an E-value of less than 1x10^-5^ was returned were considered valid. The results were post-processed, so that contigs were assembled into super-contigs according to the alignment along the species specific transcripts.

*ORF prediction* – The prediction of open-reading frames (ORFs) in the transcripts was achieved using Trinity’s longest ORF prediction method (11).

*Differential expression* – The reads of each subject were aligned to the final combined contig set using bowtie2 optimizing the search for local-alignments, and using the very-accurate approach (6). The number of reads from each sample that aligned to a given contig was determined using custom scripts. These values were then summed across all contigs that matched to the same uniprot definition; the counts were adjusted downwards to reflect the occasions when a read aligned to different contigs of the same uniprot reference thereby preventing the double-counting of each read. The differential expression analysis was performed within the R statistics environment v2.15 (R Core Team, 2013) using the EBSeq (12). Multiple testing statics (FDR and FWER) were applied via the multi-test package (13). Differential expression was determined from the respect of NTSR Vs HS populations rather than the sensitive individuals.

*Gene ontology analysis* – The GO-codes for each uniprot identifier were assigned using the biomaRt package (14), using Uniprot’s unimart interface (15). Enrichment analysis was performed using the hypergeometric calculation within R (16) where enrichment in gene sets that had a p-value less than 0.05 and showed either a 2-fold increase, or decrease, in the posterior calculations were considered significant.

*Data analysis –* Transcriptome (contig) sequences that were significantly differently expressed between the susceptible and resistant populations (fold change >2, FDR<0.05) were submitted to Mercator annotation using standard settings to assign functional BINs to contig sequences (17). This enabled visualization of functions represented by sequences throughout the transcriptome that were significantly up or down regulated in NTSR Vs. HS populations. Given that the number of contigs mapped in the NTSR plants was greater overall for all 36 BINS, normalization was applied to show the percentage of up-regulated relative to mapped contigs for each population.

**References**

1. Tetard-Jones C, Kertesz MA, Gallois P, & Preziosi RF (2007) Genotype-by-genotype interactions modified by a third species in a plant-insect system. *The American naturalist* 170(3):492-499.

2. Michel BE (1983) Evaluation of the Water Potentials of Solutions of Polyethylene Glycol 8000 Both in the Absence and Presence of Other Solutes. *Plant physiology* 72(1):66-70.

3. Altschuler M & Mascarenhas JP (1982) Heat shock proteins and effects of heat shock in plants. *Plant Molecular Biology* 1(2):103-115.

4. Li H*, et al.* (2009) The Sequence Alignment/Map format and SAMtools. *Bioinformatics* 25(16):2078-2079.

5. Quinlan AR & Hall IM (2010) BEDTools: a flexible suite of utilities for comparing genomic features. *Bioinformatics* 26(6):841-842.

6. Langmead B & Salzberg SL (2012) Fast gapped-read alignment with Bowtie 2. *Nature methods* 9(4):357-359.

7. Chevreux B*, et al.* (2004) Using the miraEST assembler for reliable and automated mRNA transcript assembly and SNP detection in sequenced ESTs. *Genome research* 14(6):1147-1159.

8. Loman NJ*, et al.* (2012) Performance comparison of benchtop high-throughput sequencing platforms. *Nat Biotech* 30(5):434-439.

9. Rothberg JM*, et al.* (2011) An integrated semiconductor device enabling non-optical genome sequencing. *Nature* 475(7356):348-352.

10. Li W & Godzik A (2006) Cd-hit: a fast program for clustering and comparing large sets of protein or nucleotide sequences. *Bioinformatics* 22(13):1658-1659.

11. Parra G, Blanco E, & Guigo R (2000) GeneID in Drosophila. *Genome research* 10(4):511-515.

12. Leng N*, et al.* (2013) EBSeq: an empirical Bayes hierarchical model for inference in RNA-seq experiments. *Bioinformatics* 29(8):1035-1043.

13. Pollard KS, Dudoit S, & van der Laan MJ (2005) Multiple Testing Procedures: the multtest Package and Applications to Genomics. *Bioinformatics and Computational Biology Solutions Using R and Bioconductor*, eds Gentleman R, Carey VJ, Huber W, Irizarry RA, & Dudoit S (Springer New York, New York, NY), pp 249-271.

14. Durinck S, Spellman PT, Birney E, & Huber W (2009) Mapping identifiers for the integration of genomic datasets with the R/Bioconductor package biomaRt. *Nature protocols* 4(8):1184-1191.

15. Magrane M & Consortium U (2011) UniProt Knowledgebase: a hub of integrated protein data. *Database: The Journal of Biological Databases and Curation* 2011:bar009.

16. Team RC (2013) R: A Language and Environment for Statistical Computing (R Foundation for Statistical Computing, Vienna).

17. Gotz S*, et al.* (2008) High-throughput functional annotation and data mining with the Blast2GO suite. *Nucleic acids research* 36(10):3420-3435.
